# The Sight-Testing Opticians Bill

**Published:** 1905-09-30

**Authors:** 


### The Hospital.

A Journal of

The Medical Sciences and Hospital Administration.

Vol. XXXVIII.—No. 992. SATURDAY, SEPTEMBER 30, 1905.

### The Sight-testing Opticians Bill.

The claims of certain opticians and spectacle makers to possess special knowledge and skill in the estimation and treatment of the refractive errors more or less frequently present in the human eye, have, of recent years, been advanced with considerable emphasis. The attempt is now to be made to secure for these claims legal recognition and sanction and to create under the authority of the law a corresponding and distinctive title, the use of which shall be restricted to those who fulfil certain conditions to be defined in a special Act of Parliament. It is proposed, in short, that the terms "Optologist" and "Sight-testing Optician" shall be presented to the public as a guarantee that the holder is a person certified by examination and recognised by legal enactment as specially qualified "to practise sight-testing." To attain these ends Parliament is to be asked to pass into law certain provisions duly set forth in the "Sight-testing Opticians Bill."

The proposals thus advanced include the creation of a General Optical Council, upon which is to fall the responsibility of forming and keeping a Register of persons qualified in accordance with the provisions of the Act. Admission to the Register may be obtained by persons who have passed examinations in sight-testing approved by the General Council, and these, in a memorandum which accompanies the Bill, are stated to be the examinations of the British Optical Association and of the Spectacle Makers' Company. On the other hand, any non-registered person shall be prohibited not only from the titles recognised by the Bill but also from the use of any other name, title, designation, addition or description which implies that "he is a person specially qualified to practise sight-testing or testing sight." That is to say, if the clauses of this Bill become law no persons, medical practitioners being specially excepted, other than those whose names appear on the Optical Register can legally profess to engage in sight-testing, whilst the terms "sight-testing optician" and "optologist" will carry a legalised guarantee of competence and efficiency.

With the exact terms and details of the Bill here summarised we have at present no special concern. It may even be allowed that, speaking generally, they are well adjusted to the end which it is hoped to attain. Our quarrel, however, is not with the details, but with the principle on which they are based. We cannot admit the claim that sight-testing can be properly presented to the public as an art which can be safely carried on by persons devoid of pathological knowledge and of clinical experience, and in view of this we can only recognise the "optologist" or "sight-testing optician" as a person who is indulging in false pretences.

There can be no difficulty in defining the standard by which the issue is to be determined. Both those who favour the policy of the proposed Bill and those who oppose it, will agree that the question in the last resort is to be decided by an appeal to the public interest and welfare. Neither opticians nor any other persons can claim any prescriptive or logical right to a monopoly of the practice of sight-testing. Whatever policy be proposed for acceptance must make its appeal, not to the interests of a class, but to the convenience and protection of the general public. And it is because these ends are, in our judgment, in conflict with the "Sight-testing Opticians Bill" that we think this measure ought to receive the opposition of the medical profession.

It is evident that there underlies the whole of these proposals the argument that there are certain defects of sight which are purely optical and others which depend on structural disease, and that the distinction between the two can be confidently made by non-medical persons who have undergone a certain technical training in the art of testing the sight. Now it may be admitted that many patients do as a matter of fact get reasonably accurate glasses without undergoing any medical examination. But it is equally certain that in not a few cases defects of sight are corrected by opticians whilst there are present in the eye structural changes that would be recognised by a medical practitioner as of the highest significance in relation to the necessary treatment and protection of the patient. In short, experience shows, what indeed was to be anticipated from the outset, that the evidences, and especially the early evidences of disease in any particular part of the body can only be recognised by those who have had an adequate training in general pathology and a wide clinical education.

Hence it is misleading the public to create and legalise a title which implies that those who exhibit this are persons competent and qualified to give advice and treatment for defects of sight. For whilst it may be true that some opticians are more ignorant than others, they are all, as professed advisers in a matter involving the recognition or exclusion of disease, tainted by the same vice—namely, absence of the discipline and experience which alone make these conclusions ready and confident. If individual members of the public are satisfied to accept guidance of this order that is a matter of personal choice, with which we have no concern. But to propose parliamentary sanction for the proceeding is entirely another matter.

Again, we maintain that defects of sight cannot, any more than other disabilities, be separated from questions concerning the general health and personal peculiarities of the individual, and the appreciation of these is certainly not to be acquired by an arbitrary training in the art of sight-testing. To imply the contrary and to affirm that certain persons who know nothing of the larger questions are fully competent to deal with the condition of the eyesight is to delude and deceive the public. Lastly, and apart from the above considerations, it has to be recognised that in many, perhaps in the majority of instances, the state of the refraction and the condition of the eye can only be successfully effected by the use of mydriatics. It can hardly be contended that these powerful agents are safe in the hands of persons trained in opticians' shops. Yet one of two positions must be accepted: Either such persons must use these agents or they must admit that it is impossible for them safely and adequately to deal with many defects which cause imperfect sight.

These are some of the reasons which warrant opposition to a proposal to set up a special class of persons with a legalised pretence to do that for which they have neither the education nor the experience. That they will appeal to members of the medical profession we have no doubt, and we count with not less confidence on the sympathies of the more intelligent members of the general public.

